# Morphometric Analysis of Rat Prostate Development: Roles of MEK/ERK and Rho Signaling Pathways in Prostatic Morphogenesis

**DOI:** 10.3390/biom11121829

**Published:** 2021-12-04

**Authors:** Wen-Yang Hu, Parivash Afradiasbagharani, Ranli Lu, Lifeng Liu, Lynn A. Birch, Gail S. Prins

**Affiliations:** 1 Department of Urology, College of Medicine, University of Illinois at Chicago, Chicago, IL 60612, USA; pafrad2@uic.edu (P.A.); ranlilu@uic.edu (R.L.); lifeng@uic.edu (L.L.); lbirch@uic.edu (L.A.B.); gprins@uic.edu (G.S.P.); 2 Chicago Center for Health and Environment, Division of Epidemiology & Biostatistics, School of Public Health, University of Illinois at Chicago, Chicago, IL 60612, USA; 3 Departments of Pathology, Physiology & Biophysics, Division of Epidemiology & Biostatistics, School of Public Health, College of Medicine, University of Illinois at Chicago, Chicago, IL 60612, USA; 4 University of Illinois Cancer Center, College of Medicine, University of Illinois at Chicago, Chicago, IL 60612, USA

**Keywords:** prostate, branching morphogenesis, FGF10, ERK1/2, Rho kinase, myosin light chain kinase, rat prostate lobes

## Abstract

The molecular mechanisms underlying prostate development can provide clues for prostate cancer research. It has been demonstrated that MEK/ERK signaling downstream of androgen-targeted FGF10 signaling directly induces prostatic branching during development, while Rho/Rho-kinase can regulate prostate cell proliferation. MEK/ERK and Rho/Rho kinase regulate myosin light chain kinase (MLCK), and MLCK regulates myosin light chain phosphorylation (MLC-P), which is critical for cell fate, including cell proliferation, differentiation, and apoptosis. However, the roles and crosstalk of the MEK/ERK and Rho/Rho kinase signaling pathways in prostatic morphogenesis have not been examined. In the present study, we used numerical and image analysis to characterize lobe-specific rat prostatic branching during postnatal organ culture and investigated the roles of FGF10-MEK/ERK and Rho/Rho kinase signaling pathways in prostatic morphogenesis. Prostates exhibited distinctive lobe-specific growth and branching patterns in the ventral (VP) and lateral (LP) lobes, while exogenous FGF10 treatment shifted LP branching towards a VP branching pattern. Treatment with inhibitors of MEK1/2, Rho, Rho kinase, or MLCK significantly inhibited VP growth and blocked branching morphogenesis, further supporting critical roles for MEK/ERK and Rho/Rho kinase signaling pathways in prostatic growth and branching during development. We propose that MLCK-regulated MLC-P may be a central downstream target of both signaling pathways in regulating prostate morphogenesis.

## 1. Introduction

Developmental biology contributes greatly to the understanding of cancer biology. Accumulating evidence indicates that cancer contains many features of development gone awry, with several developmental genes frequently reactivated in tumor tissues [1,2]. Thus, knowledge of the molecular mechanisms underlying organ development is essential for gaining novel insights into cancer growth, its progression, and the development of therapeutical intervention strategies.

Prostate development is dependent on androgens and mesenchymal/epithelial interactions [3,4,5,6]. The rodent prostate gland is the traditional model for prostate research. Different from the prostate in rodents that consists of anatomically distinct lobes, the human prostate is more compact and composed of three different histological zones. Although it is hard to make direct analogies to the human prostate, the rat prostate is larger in size and more complex histologically than the mouse prostate, making it a better model for developmental studies [7]. The rat prostate gland consists of three lobes, the ventral (VP), lateral (LP), and dorsal (DP) lobes, which exhibit intricate and unique branching patterns [8,9]. Rat prostate development starts with the formation of urogenital sinus (UGS) epithelial cells buds that penetrate the surrounding UGS mesenchyme in the ventral, dorsal, and lateral directions. At birth, the rat VP, LP, and DP lobes primarily consist of solid elongating main ducts and subsequent outgrowth occurs postnatally. At this developmental stage, ductal epithelial cells proliferate primarily at the leading edge of distal tips [9,10,11]. Ductal branching begins when the elongating epithelial main ducts interact with the surrounding mesenchymal pads peripheral to the periurethral smooth muscle, inducing secondary, tertiary, and further branching events with increasing complexity [9,10,11]. Formation of the prostate secretory ducts involves lumenization of the tubular epithelium in the proximal to distal direction, a common feature of vertebrate organogenesis [3,8]. The separate rodent prostate lobes exhibit unique branching patterns suspected to be a function of differential mesenchymal morpho-regulatory gene expression [9,10,11]; however, this remains to be clearly established. As such, comparative studies allow for delineation of the differential mechanisms whereby distinctive branching patterns arise [9,10,12].

Additional insight into prostate development comes from prostate organ culture systems that allow functional testing of a given signaling pathway [5,13,14,15,16]. However, most studies have not interrogated the branching process in detail but rather report on the final endpoints in the whole cultured VP. While a few studies have performed image analysis of prostatic growth and branching morphogenesis, numerical analysis of lobe-specific comparisons has not been elucidated in detail [10,13,17].

Prostate development is controlled through coordinated expression and crosstalk among multiple stimulatory and inhibitory regulators that include developmental genes; secreted signaling molecules; and transcription factors that are regulated by androgens, estrogens, and retinoids [13,17,18,19,20,21,22,23,24]. While androgens are essential and sufficient for prostate growth, whether androgens drive growth and branching independently of these morpho-regulatory factors remains unknown. We previously determined that androgens regulate expression of several rat prostate morpho-regulatory genes via fibroblast growth factor 10 (FGF10)-dependent and -independent pathways [24,25]. FGF10, secreted by prostate mesenchymal cells, directly induces prostatic branching through FGFR2iiib on distal tip epithelial cells, and activation of downstream signaling through MEK/ERK is required for both prostate budding and ductal branching [24,25,26,27,28].

Rho signaling has been implicated in branching morphogenesis of the mammary gland and lung [29], but its role in prostate branching has not yet been studied. Further, crosstalk of the MEK/ERK and Rho signaling pathways as well as their common downstream targets in prostatic morphogenesis regulation have not been reported. Phosphorylation of myosin light chain (MLC-P) plays a key role in cell proliferation, division, and migration. MLC-P is regulated by myosin light chain kinase (MLCK) [30,31], Ras, and Rho. Ras-activated ERK phosphorylates and stimulates MLCK activity, while Rho increases MLC-P by phosphorylating and inactivating the targeting subunit of myosin phosphatase 1 (MYPT). Therefore, MLC-P regulated by the MEK/ERK and Rho signaling pathways may play a critical role in prostate morphogenesis [32]. 

In the present study, we sought to fully elucidate the branching phenotypes of the separate prostate lobes using the rat VP and LP as models, as well as to interrogate the roles of and crosstalk between FGF10-MEK/ERK and Rho signaling pathways in regulating and determining the branching patterns. Towards this end, we utilized neonatal organ culture with numerical and real-time image analyses to delineate lobe-specific branching morphogenesis characteristics. Next, LPs were cultured with exogenous FGF10, and results show the emergence of a VP branching pattern. The roles of MEK/ERK and Rho signaling pathways in prostate morphogenesis were subsequently investigated in VP organ cultures via inhibitor-based studies. Our findings implicate critical roles of MEK/ERK and Rho/Rho kinase signaling pathways in prostatic growth and branching and highlight MLCK-regulated MLC-P as a central downstream target of both signaling pathways in regulating prostate morphogenesis. 

## 2. Materials and Methods

### 2.1. Animals

All animals were housed and handled according to the procedures of the Guiding Principles for the Care and Use of Animal Research, and all experiments were approved by the Institutional Animal Care and Use Committee (UIC Animal Welfare Assurance No. D16-00290(A3460-01)). Timed pregnant female Sprague-Dawley rats were purchased from Zivic-Miller (Pittsburgh, PA, USA), housed individually in a temperature (21 °C) and light (14 h light/10 h dark)-controlled room, and fed standard Purina rat chow (Ralston-Purina, St. Louis, MO, USA) ad libitum. They were monitored daily for delivery of pups, and the day of birth was designated as day 0. Pups were euthanized on postnatal day (pnd) 0 or 2, UGS-prostate complexes were removed, and the separate prostate lobes were micro dissected for separate organ cultures. 

### 2.2. Prostate Organ Culture and Imaging

Prostate organ culture details are described in our previous studies [17,24,25]. To examine the effects of MEK/ERK and Rho signaling pathway modulators on normal rat prostate ductal branching, we removed VP and LP lobes by microdissection on pnd 0 (within 4 h of birth), and paired, contralateral lobes from a single pup (n = 6) were cultured for 4 days in basal culture medium (BOCM) without or with 20 μM U0126 (selective MEK1/2 inhibitor), 2 μg/mL C3 transferase (Rho inhibitor), or 20 μM Y-27632 (Rho kinase inhibitor). Paired LP lobes were cultured for 4 days in the absence or presence of 1 μg/mL FGF10. For lateral prostate type 1 (LP1) and type 2 (LP2) culture, lateral prostate lobes were first dissected on pnd 2, and the LP1 and LP2 were further separated under a dissecting microscope (Nikon, East Rutherford, NJ, USA). The BOCM consisted of DMEM/F-12 (Invitrogen/GIBCO, Carlsbad, CA, USA), 50 µg/mL gentamycin, 1× insulin–transferrin–selenium (Invitrogen), and 10 nM testosterone (Sigma-Aldrich, St Louis, MO, USA), and was replaced every 48–72 h. Tissues were floated on Millicell-CM filters (Millipore Corp., Bedford, MA, USA) in 2 mL medium in BD Falcon 12-well plates (BD Biosciences, San Jose, CA, USA) inside a closed culture chamber at 37 °C with ports for introduction and exhaust of humidified 5% CO_2_ (Carl Zeiss MicroImaging, Inc., Thornwood, NY, USA).

Culture chambers were placed within a heated incubator attached to a Zeiss Axiovert 200 inverted microscope with an automated X-Y-Z stage and Axiocam HRm digital camera. Movement of the automated stage to photograph each cultured prostate in sequence and image acquisition were controlled by a computer running AxioVision 4.9.1 and Zeiss Image Pro3.0 software. Digital photographs of each sample were captured at 30 min intervals. For time-lapse imaging, still images taken over the first 96 h were converted to a QuickTime format using QuickTime Pro. Morphometry was performed on images taken at 10 h intervals from 0–90 h. A color-coded skeletal map was drawn for each duct using AxioVision software to denote the successive generations of branches from the main branch over time. The number of segments for each branch generation, the length of each segment, the total ductal length of each generation, the number of branchpoints, and the total tip number were measured. To correctly classify branching events as terminal bifed, trifed, or lateral branching, we examined additional images between the 10 h time points. Data were analyzed by analysis of variance (ANOVA) followed by Bonferroni post hoc tests (Instat ver 3.01, GraphPad Software, Inc., San Diego, CA, USA), using 6–10 samples for each treatment group.

### 2.3. Hematoxylin and Eosin (H&E) Staining

Prostatic tissues were fixed in 4% paraformaldehyde overnight, followed by dehydration, as described in our previous study [17]. Tissues were then embedded in paraffin blocks. Cross-sections (4 microns thick) were cut. Tissue sections were then deparaffinized and rehydrated for hematoxylin and eosin-y staining as described previously [17]. Stained sections were examined using a Zeiss Axioskop 20 microscope. 

### 2.4. Cell Culture 

NRP152 rat prostate epithelial cells were cultured in GM2 culture medium consisting of DMEM/F12, 5% fetal bovine serum (Gibco, Gaithersburg, MD, USA), 5 μg/mL bovine insulin, 20 ng/mL EGF, 10 ng/mL cholera toxin, and 0.1 µM dexamethasone (Sigma-Aldrich, St. Louis, MO, USA). To split the cells, culture medium was removed, cells were washed twice with PBS followed by addition of 5 mL trypsin/EDTA (Gibco, Gaithersburg, MD, USA), and incubated at 37 °C for 5–10 min. The detached cells were counted and inoculated in 96-well plates for the MTS cell proliferation assay and in 6-well plates for the MLC-P biochemistry study. 

### 2.5. MTS Cell Proliferation Assay

NRP152 cells in 96-well plates were treated with different doses of ML-7 (0, 10, 20, or 30 μM) for 24, 48, or 72 h with 4 wells/ML-7 dose at each time point. Cell proliferation activity was evaluated by the MTS cell proliferation assay using the CellTiter 96^®^AQ_ueous_ non-radioactive cell proliferation assay kit (Promega, Madison, WI, USA). Briefly, 2.0 mL MTS Solution was aseptically transferred to a test tube, and 100 μL PMS Solution was added. After gentle mixing, 20 μL of combined MTS/PMS solution was immediately added to each well of the 96-well plate containing 100 μL of cells in culture medium. After 4 h incubation at 37 °C in a humidified, 5% CO_2_ atmosphere, the absorbance at 490 nm was recorded using an ELISA plate reader. Extra wells of cells without the addition of MTS/PMS solution were used as background for calibration. Average relative OD values were then calculated. 

### 2.6. MLC-P Measurement by Western Blot

NRP152 cells in 6-well plates were treated with 20 μM U0126, 20 μM Y27632, and 10 μM ML-7 (MLCK inhibitor) for 24 h. MLC-P was quantified using a urea/glycerol gel-immunoblotting method as described in our previous studies [26,27]. Briefly, cells were fixed in ice-cold 10% trichloroacetic acid (TCA) containing 10 mM dithiothreitol (DTT) (Sigma-Aldrich, St. Louis, MO, USA). Cells were collected using a cell lifter and pelleted by centrifugation at 2000× *g* for 8 min. After washing four times with acetone to remove TCA, the cell pellets were dissolved in buffer containing 9 M urea, 10 mM DTT, and 20 mM Tris (pH 8.0) for protein extraction. Protein was separated using urea/glycerol polyacrylamide gel electrophoresis. The proteins were transferred to nitrocellulose and probed with an affinity-purified antibody to MLC_20_ (Gift from Dr. Primal de Lanerolle, University of Illinois at Chicago). This antibody recognizes the un-, mono-, and di-phosphorylated forms of MLC_20_. Immunoreactive bands were visualized using enhanced chemiluminescence detection reagents (Amersham-Pharmacia Biotech, Piscataway, NJ, USA), and the stoichiometry of phosphorylation of MLC (mol PO_4_/mol MLC_20_) was calculated.

### 2.7. Statistical Analysis

All data are expressed as means ± SEM. The data were analyzed using Student’s *t*-test or, for multiple measures, one-way ANOVA (SigmaStat, Systat Software Inc., Palo Alto, CA, USA) followed by post hoc tests. Values of *p* < 0.05 were considered to be significant.

## 3. Results

### 3.1. Prostates Exhibit Distinguishing Lobe-Specific Growth and Branching Patterns

**Figure 1:**
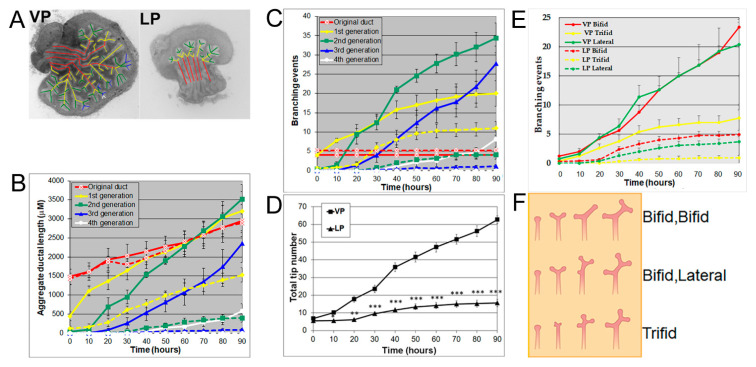
(**A**) Branch generations of pnd 0 cultured rat VP (left) and LP (right) lobes. Successive images were taken every 30 min for 90 h to track branching events. Representative end-point images at 90 h are shown. A color-coded skeleton is used to indicate branch generations according to the following convention: the primary buds emerging from the UGS are considered the original ducts (red); branches that form off the primary ducts are considered the first generation (yellow); branches that form off the first generation and elongate are considered the second generation (green), third generation (blue), and fourth generation (white). Note that VPs formed branches up to the fourth generation, while LPs stopped branching at the second generation. (**B**) Kinetics of prostatic bud and ductal elongation and successive branch generations in pnd 0 rat VPs and LPs cultured for 90 h. The aggregate length (µm) of all ductal segments in each successive branch generation over time is shown. Solid lines represent VP data and dashed lines represent LP data. (**C**) Total number of branch point events in each ductal generation as a function of time. Solid lines represent VP data and dashed lines represent LP data. Error bars denote SEM from 5 sets of experiments. Differences between the two groups are statistically significant (*p* < 0.05) from 40 h onward except for the original ducts. (**D**) Total tip number in 3D analysis as a function of time in rat pnd 0 VPs (squares) and LPs (triangles) cultured for 90 h. Each data point represents the mean of 5 sets of experiments and bars denote SEM. * denotes significant differences between VPs and LPs, * *p* < 0.05, ** *p* < 0.01, *** *p* < 0.001. (**E**) Total counts of specific branching types (terminal bifeds, terminal trifeds, lateral side branches) occurring over time in VPs vs. LPs. Each data point represents the mean of 5 sets of experiments, and bars denote SEM. Differences between VPs and LPs are statistically significant (*p* < 0.05) from 30 h onward. (**F**) Illustration of three branching types: terminal bifeds, terminal trifeds, and lateral side branches. The branching schematic at the far right is identical in each row. However, they each came about through 3 different sequences of branching events over time: bifed-bifed, bifed-lateral, and trifed branching. Only sequence imaging over time can correctly clarify the specific branching events.

Using a numerical and image analysis system developed in our lab [13,17], we evaluated lobe-specific prostatic branching in the neonatal VP and LP cultured for four days. At pnd 0, each VP lobe had 3–5 long main ducts emanating from the UGS, while each LP had 5–6 shorter main ducts. Starting from the original ducts, the VP formed branches up to the fourth generation, resembling an elm tree, whereas the LP stopped branching at the second generation, resembling a bushy tree (Figure 1A), descriptors previously used by Hayashi et al. [13]. Compared to the LP, the VP had a significantly longer aggregate ductal segment lengths in each successive branch generation after the original duct over time (Figure 1B) and more total branching events from the original ducts (Figure 1C). As a result, the VP had a higher total tip number over time compared to the LP from 10 h onward (Figure 1D). Branching events were characterized as they occurred over 30 min intervals; terminal bifeds and lateral branches predominated in the VP with terminal trifeds as a minor percent (Figure 1E,F). In the LP, terminal bifeds were the dominant branch form followed by lateral branching and infrequent terminal trifeds. 

### 3.2. LP1 Had a Longer Original Ductal Length but Fewer Branching Events and Fewer Tips Compared to LP2

**Figure 2:**
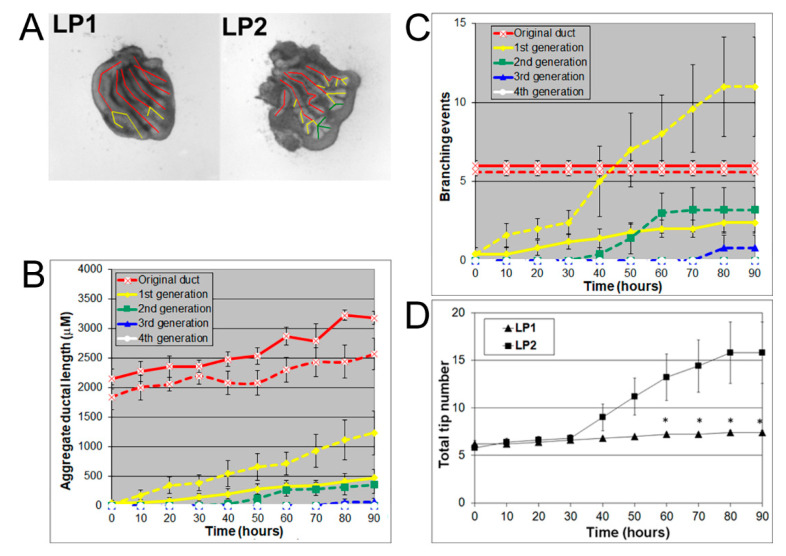
(**A**) Branch generations of cultured contralateral rat day 2 LP1 (left) and LP2 (right) lobes. Successive images were taken every 30 min for 90 h to track branching events. A color-coded skeleton is used to denote the branch generations as described in Figure 1. Representative end-point images are shown at 90 h. Note that LP1s have longer original ducts and formed branches only up to the first generation over the 4-day culture, whereas LP2s have shorter original ducts and formed branches up to the third generation. (**B**) Kinetics of prostatic bud and duct elongation over successive branch generations in pnd 2 rat LP1s and LP2s cultured for 90 h. The aggregate length (μm) of all ductal segments in each successive branch generation over time is shown. Solid lines represent data from LP1s, and dashed lines represent data from LP2s. (**C**) Total number of branch point events in each ductal generation as a function of time. Solid lines represent data from control LP1s, and dashed lines represent data from LP2s. Error bars denote SEM from 5 sets of experiments. Differences between the two groups were statistically significant (*p* < 0.05) from 20 h onward except for the original ducts. (**D**) Total tip number in 3D analysis as a function of time in rat day 2 LP1s (squares) and LP2s (triangles) cultured for 90 h. Each data point represents the mean of 5 sets of experiments and bar denotes SEM. * denotes significant differences between LP1s and LP2s, *p* < 0.05.

At pnd 2, the rat lateral prostate can be further separated into two functionally different subtypes: type 1 (LP1) and type 2 (LP2) lateral prostates. The LP1 extends cranially towards the seminal vesicle and DP, and the LP2 arborizes caudal to the bladder neck and gives rise to compact bushy glands [13]. Hayashi et al. showed that the major secretory proteins of LP1 are DP1 and DP2 proteins, while the major secretory protein of LP2 is probasin [9,10,12]. In the current study, LP1 and LP2 were carefully separated into their individual ductal sub lobes as they emerged from the UGS at pnd 2 and cultured for four days with successive images taken every 30 min for 90 h to track branching events. LP1 and LP2 both had 5–6 original ducts budding from the UGS. LP1 had longer original ducts and formed branches only up to the first generation, resembling a miniature palm tree, whereas LP2 had shorter original ducts and formed branches up to the third generation, resembling a bush (Figure 2A). Numerical and image analysis showed that the aggregate ductal length of LP1 was longer in the original ducts but shorter in the first generation compared to LP2 (Figure 2B). LP1 also had fewer branching events (Figure 2C) and fewer total tips (Figure 2D) than LP2. 

### 3.3. Effects of MEK Inhibition and FGF10 Supplementation on Ventral or Lateral Prostate Growth and Branching Morphogenesis

**Figure 3:**
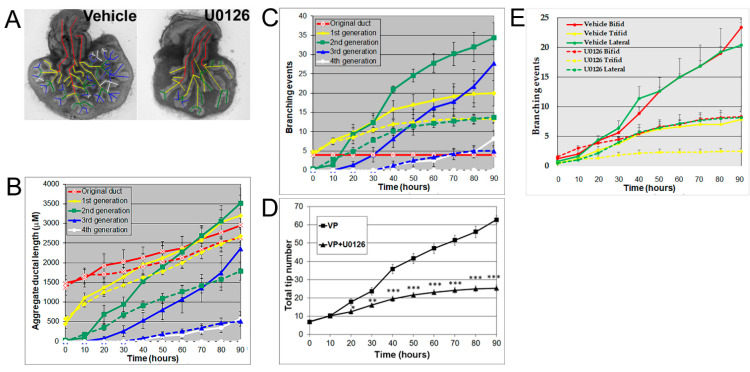
(**A**) Branch generation in contralateral rat pnd 0 VPs cultured for 90 h in the absence (left) or presence of 20 μM U0126 (right). Successive images were taken every 30 min for 90 h to track branching events. A color-coded skeleton is used to indicate branch generation, as described in Figure 1. Representative end-point images at 90 h are shown. Note that untreated VPs formed branches up to the fourth generation, whereas U0126 treatment blocked the formation of branches at the third generation. (**B**) Kinetics of prostatic bud and duct elongation over successive branch generations in pnd 0 VPs cultured for 90 h in the absence (solid lines) or presence of 20 μM U0126 (dashed lines). The aggregate length (μm) of all ductal segments in each successive branch generation over time is shown. (**C**) Total number of branch point events in each ductal generation as a function of time. Solid lines represent data from untreated VPs, and dashed lines represent data from U0126-treated VPs. Error bars denote SEM from 5–7 sets of experiments. Differences between the two groups were statistically significant (*p* < 0.05) from 50 h onward, except for the original ducts. (**D**) Total tip number in 3D analysis as a function of time in rat contralateral pnd 0 untreated VPs (squares) and U0126-treated VPs (triangles) cultured for 90 h. Each data point represents the mean of 5–7 sets of experiments and bars denote SEM. * denotes significant differences between two groups, * *p* < 0.05, ** *p* < 0.01, *** *p* < 0.001. (**E**) Total counts of branching events occurring as terminal bifeds, terminal trifeds, or lateral side branches in VPs cultured in the absence or presence of 20 µM U0126. Differences between the vehicles and U0126 treatments are statistically significant (*p* < 0.05) from 50 h onward.

Androgens are essential for prostate development, with morphogenesis driven by androgen-dependent, mesenchymal-derived, paracrine-acting factors, including FGF10 [24,25,28], which acts through MEK/ERK signaling [25,27]. The MEK/ERK signaling pathway was confirmed for the present studies using U0126, a small molecule inhibitor of MEK1/2 activity, the kinase responsible for phosphorylating ERK1/2. Treatment with 20 μM U0126 significantly inhibited VP growth and branching morphogenesis, shifting its pattern towards an LP phenotype (Figure 3A) (Supplemental Video). Numerically, the decrease of total ductal length by U0126 showed an inhibition of ductal elongation (Figure 3B), while the decrease in branching events (Figure 3C) and total tip number (Figure 3D) by U0126 documented the inhibitory effect on prostate branching to LP-type patterns. These results implicate important roles of MEK/ERK signaling in both ductal elongation and prostate branching and suggest that these separate processes are controlled by the same signaling pathway. In control VP cultures, 42.3% of branches were terminal bifid, 19.7% were terminal trifid, and 38% were lateral branch points. U0126 treatment had no significant effect on the distribution pattern of branching types (Figure S1), blocking all three branching types to a similar degree (Figure 3E). An increase in ductal thickness with U0126 treatment indicates that some degree of epithelial proliferation continued. 

**Figure 4:**
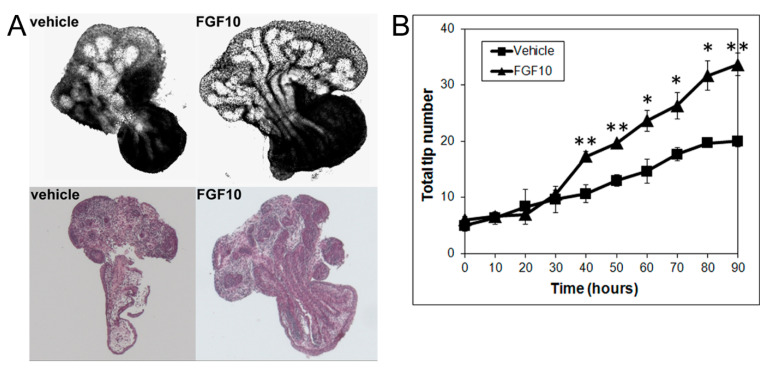
(**A**) Branch generations of contralateral rat pnd 0 LPs cultured for 90 h in the absence (left) or presence of 1 µg/mL FGF10 (right). Representative end-point images at 90 h are shown (upper panel). H&E staining of corresponding tissues are also shown (lower panel). Note the increased branch formation in 1 µg/mL FGF10-treated LPs. (**B**) Total tip number in 3D analysis as a function of time in contralateral pnd 0 untreated LPs (squares) and 1 µg/mL FGF10-treated LPs (triangles) cultured for 90 h. Each data point represents the mean of 4 sets of experiments, and bars denote SEM. * denotes significant differences between vehicle and FGF10 treatment, * *p* < 0.05, ** *p* < 0.01.

Since the present results using image and numerical morphometry showed that blocking FGF10 downstream signaling using a MEK1 inhibitor reduced VP growth and branching morphogenesis and shifted the branching pattern towards a LP phenotype, we next examined the effects of exogenous FGF10 on LP morphogenesis. As predicted, FGF10 (1 µg/mL) supplementation to LP cultures significantly stimulated LP prostate growth and increased the total branching events towards a VP branching pattern (Figure 4).

### 3.4. Effects of Rho, ROCK, and MLCK Inhibitors on Ventral Prostate Growth and Branching Morphogenesis

**Figure 5:**
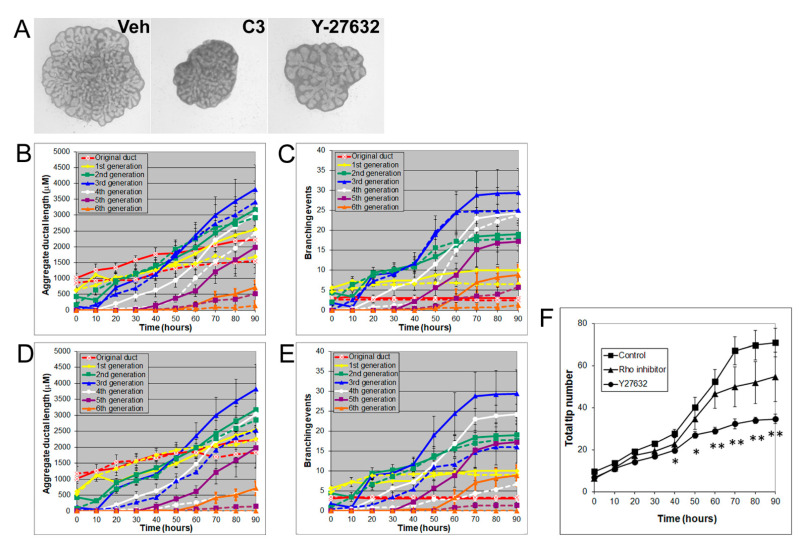
(**A**) Branch generations of contralateral rat pnd 0 VPs cultured for 90 h with vehicle control (left), 2 µg/mL C3 transferase (middle), or 20 µM Y-27632 (right). Successive images were taken every 30 min for 90 h to track branching events. Representative end-point images at 90 h are shown. Note that there were fewer branch formations in the second to fifth generation with both C3 transferase and Y-27632 treatments. (**B**) Kinetics of prostatic bud and duct elongation over successive branch generations in pnd 0 VPs cultured for 90 h in the absence (solid lines) or presence of 2 µg/mL C3 transferase (dashed lines). The data show the aggregate length (µm) of all ductal segments in each successive branch generation over time. (**C**) Total number of branch point events in each ductal generation as a function of time. Solid lines represent data from untreated VPs, and dashed lines represent data from 2 µg/mL C3-transferase-treated VPs. Error bars denote SEM from 4 sets of experiments. Differences between the two groups were statistically significant (*p* < 0.05) from 20 h onward, except for the original ducts. (**D**) Kinetics of prostatic bud and duct elongation over successive branch generations in pnd 0 VPs cultured for 90 h in the absence (solid lines) or presence of 20 µM Y-27632 (dashed lines). Data show the aggregate length (µM) of all ductal segments in each successive branch generation over time. (**E**) Total number of branch point events in each ductal generation as a function of time. Solid lines represent data from untreated VPs, and dashed lines represent data from 20 µM Y-27632-treated VPs. Error bars denote SEM from 4 sets of experiments. Differences between the two groups were statistically significant (*p* < 0.05) from 20 h onward except for the original ducts. (**F**) Total tip number in 3D analysis as a function of time in rat pnd 0 untreated VPs (squares), 2 µg/mL C3 transferase-treated VPs (triangles), and 20 µM Y-27632-treated VPs (circles) cultured for 90 h. Each data point represents the mean of 4 sets of experiments, and bars denote SEM. * denotes significant differences between control and treatments, * *p* < 0.05, ** *p* < 0.01.

Rho/Rho-kinase signaling regulates branching morphogenesis in the mammary gland and lung [29]. To investigate the role of Rho/Rho kinase signaling in prostate morphogenesis, we treated the VP with inhibitors of Rho (C3 transferase, 2 µg/mL) or Rho kinase (Y27632, 20 µM). Both C3 transferase and Y27632 significantly inhibited VP growth and branching by decreasing the total ductal length, branching events, and total tip number, indicating an important role of Rho/Rho kinase signaling in prostate growth (Figure 5). Interestingly, the inhibitory effect of Y27632 on prostate ductal branching was greater than that of C3 transferase and produced thicker prostate ducts, such as U0126 treatment effects. 

**Figure 6:**
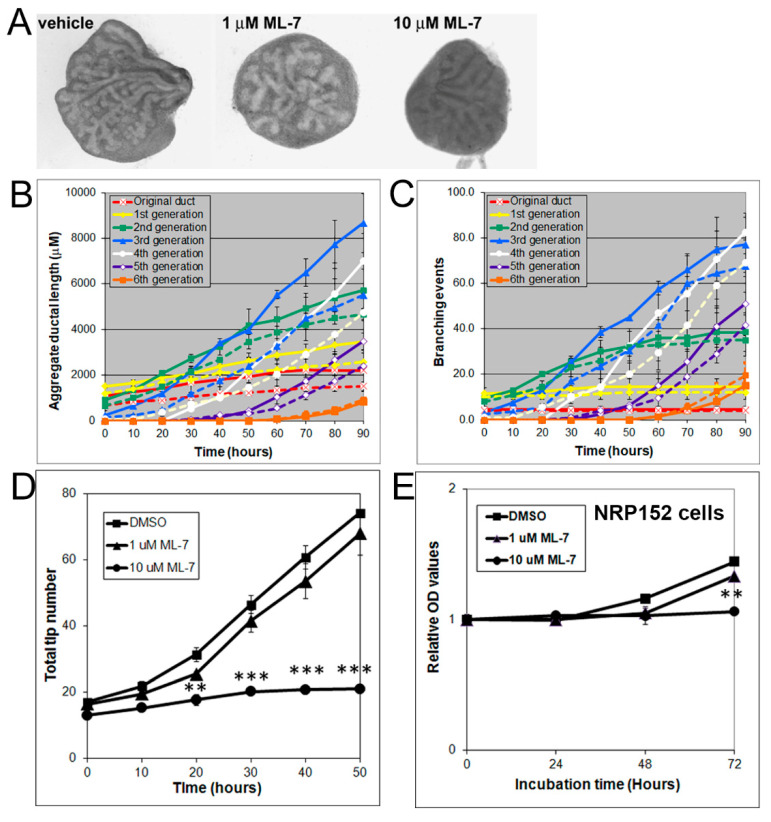
(**A**) Branching of rat pnd 0 VPs cultured for 40 h in the absence (left) or presence (middle and right) of 1–10 µM ML-7. Successive images were taken every 30 min for 90 h to track branching events. Representative end-point images at 90 h are shown. Note that ML-7 dose-dependently inhibited branch formation in VPs. (**B**) Kinetics of prostatic bud and duct elongation over successive branch events in pnd 0 VPs cultured for 40 h in the absence (solid lines) or presence (dashed lines) of 10 µM ML-7. Data show the aggregate length (µm) of all ductal segments in each successive branch generation over time. (**C**) Total number of branch point events in each ductal generation as a function of time. Solid lines represent data from untreated VPs, and dashed lines represent data from 10 µM ML-7 treated VPs. Error bars denote SEM from 4 sets of experiments. Differences between the two groups were statistically significant (*p* < 0.05) from 20 h onward except for the original ducts. (**D**) Total tip number in 3D analysis as a function of time in rat contralateral day 0 untreated VPs (squares) and ML-7 treated VPs (1 µM triangles, 10 µM circles) cultured for 40 h. Each data point represents the mean of 4 sets of experiments, and bars denote SEM. * denotes significant differences between controls and treatments, ** *p* < 0.01, *** *p* < 0.001. (**E**) Effects of an MLCK inhibitor on proliferation of cultured NRP152 rat prostate epithelial cells. NRP152 cells were treated with different doses of ML-7 (1, 10 µM) for 24, 48, or 72 h. Cell proliferation activity was evaluated by an MTS cell proliferation assay using the CellTiter 96^®^AQ_ueous_ non-radioactive cell proliferation assay kit. * denotes significant differences between control and treatments, ** *p* < 0.01, N = 4.

MLC-P, which is regulated by MLCK, plays an important role in cell proliferation, division, and migration [30,31]. Herein, we investigated whether the MLCK inhibitor ML-7, which inhibits MLC-P, can alter prostate branching morphogenesis. Indeed, 1 to 10 µM of ML-7 dose-dependently inhibited VP growth (Figure 6A) and blocked branching morphogenesis, as denoted by decreases in total ductal length, branching events, and tip numbers (Figure 6B–D). Of the inhibitors used in this study, ML-7 had the greatest inhibitory effects, and C3 transferase had the smallest effects (Figure S2). Representative H&E images from histological sections further support the inhibitory effects of U0126, C3 transferase, Y27632 and ML-7 on VP ductal branching toward a LP branching pattern (Figure S3).

Since prostate ductal branching involves proliferation of epithelial cells, the inhibitory effects of ML-7 on prostate growth and branching in organ culture may be mediated by inhibition of prostate epithelial cell proliferation. We therefore further evaluated the effect of ML-7 on the proliferation of the rat prostate benign epithelial cell line NRP152 using an MTS proliferation assay. ML-7 at doses of 10 µM significantly inhibited NRP152 cell proliferation (Figure 6E).

### 3.5. Inhibition of Cell Proliferation and MLC-P by Inhibitors of MLCK, MEK/ERK, and Rho Kinase 

**Figure 7:**
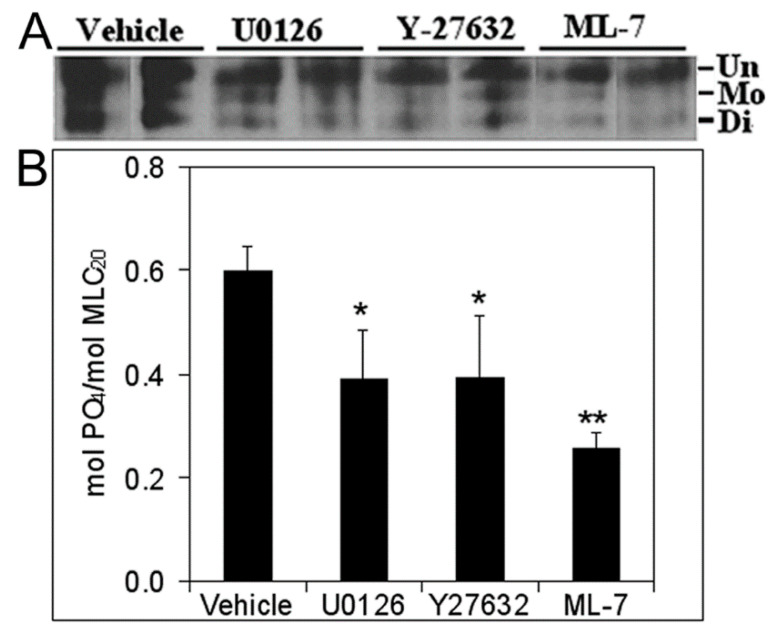
Effects of MEK/ERK and Rho signal pathway inhibitors on MLC-P in rat prostate epithelial NRP152 cells. NRP152 cells were treated with 20 µM U0126, 20 µM Y27632, and 10 µM ML-7 for 24 h. MLC-P levels were quantified by separating the unphosphorylated, mono-phosphorylated, and di-phosphorylated forms of MLC_20_ using urea/glycerol polyacrylamide gel electrophoresis followed by immunoblotting. Data from representative experiments are shown in (**A**). The stoichiometry of MLC-P (mean ± SE) is shown in (**B**). * *p* < 0.05, ** *p* < 0.01 vs. vehicle, N = 4.

MLCK-mediated MLC phosphorylation plays an important role in cell proliferation. ML-7 decreases MLC-P by inhibiting MLCK, suggesting that signaling inhibitors may inhibit cell proliferation via downregulation of MLC phosphorylation [30,31]. Importantly, both MEK/ERK and Rho signaling pathways regulate MLC phosphorylation. To further clarify whether inhibition of prostatic branching morphogenesis observed by individually targeting these two signaling pathways was mediated by MLC-P, we further examined the effects of the MEK/ERK and Rho signaling inhibitors on MLC-P in NRP152 cells by urea-glycerol gel immunoblotting. MLC can be phosphorylated into mono- and di-phosphorylation forms. About 59.7% of total MLC was phosphorylated in NRP152 cells. Treatment with 20 mM U0126, 20 mM Y-27632, or 10 mM ML-7 significantly decreased MLC phosphorylation (mol PO_4_/mol MLC_20_: 39.1% with U0126, 39.4% with Y-27632, and 25.5% with ML-7 treatment) (Figure 7). These results suggest that MLC-P is a common target of MLCK, MEK/ERK, and Rho signaling, and thus may play a central role in prostate branching morphogenesis. 

## 4. Discussion

In rodents, prostate development can be divided into five continuous stages involving determination, initiation or budding, branching morphogenesis, differentiation, and pubertal maturation. Prostate determination occurs prior to clear morphological changes and involves signaling molecules that commit a specific area of UGS cells to a prostatic cell fate [3]. There has been considerable interest in investigating the molecules and pathways that regulate prostate ductal morphogenesis due to their relevance in prostatic disease. Few studies have focused directly on prostatic branching, although some have identified molecules or pathways that play a role in prostatic growth and branching morphogenesis [3,5,6,7,9,10,11]. Several key regulators have been identified that appear to coordinately influence prostate growth and branching morphogenesis under the control of hormones and among them, developmental genes and transcription factors are most studied [3,5,6,7,9,10,11,17,21,33,34,35]. The list of signaling molecules implicated in prostatic branching morphogenesis will continue to grow as known factors cannot fully explain the extent and pattern of branching in the prostate. An emerging area of study is the crosstalk among distinct signaling pathways to determine their relative importance and hierarchy in branching regulation. Our study is the first to interrogate the Rho/Rho-kinase signaling pathway and its convergence with the FGF10-MEK/ERK pathway to phosphorylate MLC, thus identifying MLC-P as a central downstream molecule in regulating prostate development.

In the current study, by using a numerical and image analysis system developed in our lab, we compared lobe-specific prostatic morphogenesis patterns between the VP and LP in an organ culture system in the presence of testosterone [13,17,24,25]. Different lobes of the rat prostate exhibit differential sensitivity to androgens, with the ventral lobe showing stronger androgen responsiveness than the dorsal or lateral lobes [36,37]. Additionally, androgen receptor expression is highly regulated by androgens in the VP but does not respond to androgen in the LP [38,39]. The present results quantified the lobe-specific growth and branching patterns in a temporal manner. The VP exhibited increased total ductal length, branching events, and total tip number as compared to the LP. Treatment with different doses of U0126, C3 transferase, Y-27632, or ML-7 significantly reduced VP branching morphogenesis by decreasing total ductal length, branching events, and total tip number, and in so doing, shifted branching towards a LP pattern. While several studies from other groups investigated the roles of MEK/ERK and Rho-kinase in ductal branching of the prostate, lung, kidney, and other organs [40,41,42,43,44], the present study is the first to interrogate MEK/ERK, Rho kinase, and MLCK signaling in lobe-specific prostate ductal branching patterns. Furthermore, treatment with U0126, Y-27632, or ML-7 significantly decreased MLC-P and dependently inhibited cell proliferation, indicating a central role of MLCK/MLC-P in regulating prostate branching morphogenesis. 

Wnt proteins are another group of morpho-regulatory factors that influence prostate development [3,16,45,46]. We have previously shown that crosstalk between Wnt5a and Shh plays an essential role in prostate development by coordinately regulating bud outgrowth, ductal elongation, branching, and lumenization [16]. Other studies have shown that Wnt signaling can activate Rho kinase through both canonical and non-canonical pathways [47,48]. Rho/Rho-kinase integrate Wnt-induced signals spatially and temporally to promote transcriptional and morphological changes in cells regulating multiple cellular processes, including cell polarity, proliferation, differentiation, and apoptosis [49]. Our findings in the current study suggest that activation of Rho signaling pathways by Wnt ligands may be an essential downstream mechanism of prostate branching morphogenesis. In both the rodent and human, different lobes/zones of prostate exhibit functional differences in terms of the secretory and branching patterns [8]. Among the three distinct lobes of the prostate, the LP is considered to best resemble the human prostate embryologically and histologically [50]. Our previous studies in rat hormonal prostatic carcinogenesis models demonstrated a higher incidence of ductal adenocarcinoma lesions in the LP/DP compared to the VP [51,52], supporting the impact of developmental origin on carcinogenic events later in adults. Although the LP is rudimentary in the mouse, the rat LP is complex, with distinctive LP1 and LP2 branched structures. In the current study, we temporally and numerically confirmed the longer original ductal length in the LP1 with fewer branching events and fewer tip numbers compared to the LP2. Further investigation into the detailed molecular mechanisms driving the different morphogenic events during development may bring clues to delineate the underlying signals that underpin these distinct branching patterns. We and others have documented the important role of FGF10 in prostate ductal branching using the VP as the model lobe [24,25,26,27]. Herein, we further determined that differential FGF10 signaling activity underlies, in part, the different VP and LP branching patterns with higher FGF10 levels driving a VP phenotype in LPs and MEK1 inhibition driving an LP phenotype in cultured VPs. Interestingly, our previous studies on lobe-specific expression of FGF10 and FGFR2iiib found only modestly higher FGF10 levels in the VP compared to the LP and DP, while FGFR2iiib expression was lowest in the VP [25]. Although not identified in parallel wholemount in situ hybridization studies, it is possible that FGF10 localization differences along the ducts and at the distal tips in the separate lobes contributes to unique FGF10-driven branching patterns in vivo. Alternatively, differential patterning in prostate lobes may be a function of different combinations and levels of morpho-regulatory factors shown to be regulated by FGF10 that includes Shh, ptc, Bmp7, Bmp4, Wnt 2, Hoxb13, and Nkx3.1 [24,25].

**Figure 8:**
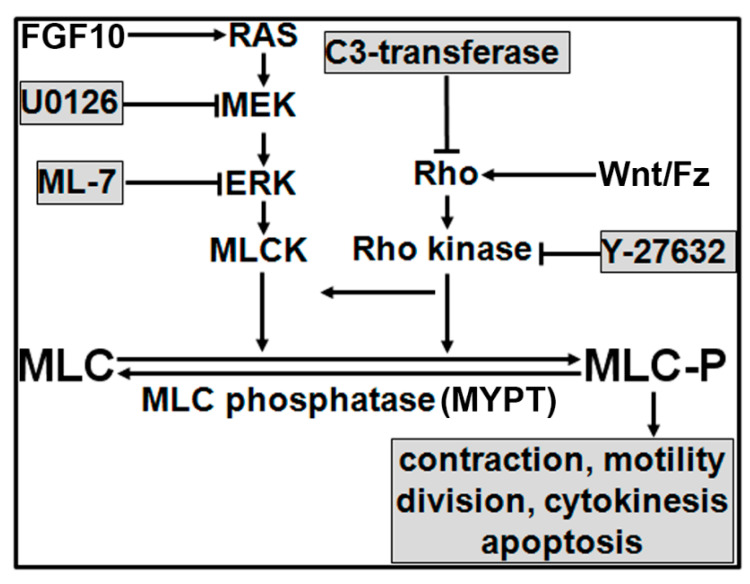
Working model. MLC-P plays a central role in cell proliferation, division, and migration, and MLCK is a dedicated kinase for MLC phosphorylation. Ras activation by FGF10 increases MLCK activity through MEK-activated ERK that phosphorylates MLCK. Rho, in contrast, increases MLC-P by phosphorylating and inactivating the targeting subunit of MYPT, which dephosphorylates MLC-P.

Data from current studies support our hypothesis that MLCK-regulated MLC-P appears to play a central role in cell proliferation, division, and migration. Our working model, shown in Figure 8, proposes that androgen stimulates mesenchymal FGF10 expression and secretion that, upon binding FGFR2iiib on epithelial cells, activates a RAS/MEK/ERK signaling cascade that increases MLCK activity. MLCK is a dedicated kinase that phosphorylates MLC. Separately, Wnt/Fz-activated Rho/Rho kinase indirectly regulates MLC-P by phosphorylating and inactivating the targeting subunit of MLK phosphatase, i.e., MYPT [30,31]. MLC-P, the common downstream target of both signaling pathways, may thus play a central role in the regulation of prostate branching morphogenesis. Blocking different signaling pathways by inhibition of MEK, MLCK, Rho, or Rho kinase using U0126, ML-7, C3-transferse, or Y-27632 may subsequently decrease MLC-P to different degrees, which in turn inhibits prostate branching morphogenesis. As aberrant branching morphogenesis occurs during localized prostate carcinogenesis, interruption of these convergent pathways may provide therapeutic benefit.

## 5. Conclusions

We herein developed a novel and useful numerical and image analysis system for evaluating prostatic morphogenesis. Rat prostates exhibit distinct lobe-specific growth and branching patterns. MEK/ERK and Rho/Rho-kinase signaling pathways are clearly involved in prostatic growth and branching morphogenesis as their inhibition prevents epithelial proliferation and consequent branching events. Further, MLCK-regulated MLC-P appears to be a central downstream target of both signaling pathways in regulating prostate morphogenesis. Androgen-regulated FGF10 signaling shifts the LP branching pattern toward a VP phenotype by increasing the formation of branches through activation of the MEK/ERK signaling pathway. In contrast, MEK inhibition results in an LP branching pattern in the VP. Together, this suggests that differential FGF10 signaling contributes to the distinctive branching patterns in these separate prostate lobes.

## Data Availability

Not applicable.

## Supplementary Materials

The following are available online at https://www.mdpi.com/article/10.3390/biom11121829/s1. QuickTime movie of contralateral VP lobes from the same newborn rat cultured in the absence (left) or presence of 20 µM U0126 (right) over 90 h. Representative videos from six separate sets of experiments are shown. Figure S1: Percentage of branching events occurring as terminal bifeds, terminal trifeds or lateral side branches in rat VPs cultured in the absence or presence of 20 µM U0126. Bars represent the mean ± SEM for 5–7 sets of experiments. Figure S2: Total tip number in 3D analysis as a function of time in rat contralateral pnd 0 untreated VPs (dark blue), 20 µM U0126 (red), C3-transferase (light blue) and 20 µM Y-27632 treated VPs (purple) as well as LPs (yellow) cultured for 90 h. Each data point represents the mean of 4 sets of experiments and bars denote SEM. Figure S3: Representative images of H&E staining from cultured rat VPs with different treatments and LP cross sections.

## References

[1] Manzo G. (2019). Similarities Between Embryo Development and Cancer Process Suggest New Strategies for Research and Therapy of Tumors: A New Point of View. Front. Cell Dev. Biol..

[2] Ma Y., Zhang P., Wang F., Yang J., Yang Z., Qin H. (2010). The relationship between early embryo development and tumorigenesis. J. Cell. Mol. Med..

[3] Prins G.S., Putz O. (2008). Molecular signaling pathways that regulate prostate gland development. Differentiation.

[4] Prins G.S., Cooke P.S., Birch L., Donjacour A.A., Yalcinkaya T.M., Siiteri P.K., Cunha G.R. (1992). Androgen receptor expression and 5 alpha-reductase activity along the proximal-distal axis of the rat prostatic duct. Endocrinology.

[5] Vickman R.E., Franco O.E., Moline D.C., Vander Griend D.J., Thumbikat P., Hayward S.W. (2020). The role of the androgen receptor in prostate development and benign prostatic hyperplasia: A review. Asian J. Urol..

[6] Cunha G.R., Donjacour A.A., Cooke P.S., Mee S., Bigsby R.M., Higgins S.J., Sugimura Y. (1987). The endocrinology and developmental biology of the prostate. Endocr. Rev..

[7] Price D. (1963). Comparative aspects of development and structure in the prostate. Natl. Cancer Inst. Monogr..

[8] Thomson A.A., Marker P.C. Branching Morphogenesis of the Prostate. Madame Curie Bioscience Database [Internet].

[9] Hayashi N., Sugimura Y., Kawamura J., Donjacour A.A., Cunha G.R. (1991). Morphological and functional heterogeneity in the rat prostatic gland. Biol. Reprod..

[10] Timms B.G., Mohs T.J., Didio L.J. (1994). Ductal budding and branching patterns in the developing prostate. J. Urol..

[11] Cunha G.R., Ricke W., Thomson A., Marker P.C., Risbridger G., Hayward S.W., Wang Y.Z., Donjacour A.A., Kurita T. (2004). Hormonal, cellular, and molecular regulation of normal and neoplastic prostatic development. J. Steroid Biochem. Mol. Biol..

[12] Thomson A.A., Marker P.C. (2006). Branching morphogenesis in the prostate gland and seminal vesicles. Differentiation.

[13] Huang L., Pu Y., Hepps D., Danielpour D., Prins G.S. (2007). Posterior Hox gene expression and differential androgen regulation in the developing and adult rat prostate lobes. Endocrinology.

[14] Lipschutz J.H., Samid D., Cunha G.R. (1996). Phenylacetate is an inhibitor of prostatic growth and development in organ culture. J. Urol..

[15] Lipschutz J.H., Foster B.A., Cunha G.R. (1997). Differentiation of rat neonatal ventral prostates grown in a serum-free organ culture system. Prostate.

[16] Martikainen P., Suominen J. (1983). A morphometric analysis of rat ventral prostate in organ culture. Anat. Rec..

[17] Huang L., Pu Y., Hu W.-Y., Birch L., Luccio-Camelo D., Yamaguchi T., Prins G.S. (2009). The role of Wnt5a in prostate gland development. Dev. Biol..

[18] Jarred R.A., Cancilla B., Prins G.S., Thayer K.A., Cunha G.R., Risbridger G.P. (2000). Evidence that estrogens directly alter androgen-regulated prostate development. Endocrinology.

[19] Prins G.S. (1992). Neonatal estrogen exposure induces lobe-specific alterations in adult rat prostate androgen receptor expression. Endocrinology.

[20] Omoto Y. (2008). Estrogen receptor-alpha signaling in growth of the ventral prostate: Comparison of neonatal growth and postcastration regrowth. Endocrinology.

[21] Huang L., Pu Y., Alam S., Birch L., Prins G.S. (2004). Estrogenic regulation of signaling pathways and homeobox genes during rat prostate development. J. Androl..

[22] Prins G.S., Chang W.Y., Wang Y., van Breemen R.B. (2002). Retinoic acid receptors and retinoids are up-regulated in the developing and adult rat prostate by neonatal estrogen exposure. Endocrinology.

[23] Vezina C.M., Allgeier S.H., Fritz W.A., Moore R.W., Strerath M., Bushman W., Peterson R.E. (2008). Retinoic acid induces prostatic bud formation. Dev. Dyn..

[24] Pu Y., Huang L., Birch L., Prins G.S. (2007). Androgen regulation of prostate morphoregulatory gene expression: Fgf10-dependent and -independent pathways. Endocrinology.

[25] Huang L., Pu Y., Alam S., Birch L., Prins G.S. (2005). The role of Fgf10 signaling in branching morphogenesis and gene expression of the rat prostate gland: Lobe-specific suppression by neonatal estrogens. Dev. Biol..

[26] Donjacour A.A., Thomson A.A., Cunha G.R. (2003). FGF-10 plays an essential role in the growth of the fetal prostate. Dev. Biol..

[27] Kuslak S.L., Marker P.C. (2007). Fibroblast growth factor receptor signaling through MEK-ERK is required for prostate bud induction. Differentiation.

[28] Thomson A.A., Cunha G.R. (1999). Prostatic growth and development are regulated by FGF10. Development.

[29] Terunuma A., Limgala R.P., Park C.J., Choudhary I., Vogel J.C. (2010). Efficient procurement of epithelial stem cells from human tissue specimens using a Rho-associated protein kinase inhibitor Y-27632. Tissue Eng. Part A.

[30] Gu L.-Z., Hu W.-Y., Antic N., Mehta R., Turner J.R., de Lanerolle P. (2006). Inhibiting myosin light chain kinase retards the growth of mammary and prostate cancer cells. Eur. J. Cancer.

[31] Fazal F., Gu L., Ihnatovych I., Han Y., Hu W., Antic N., Carreira F., Blomquist J.F., Hope T.J., Ucker D.S. (2005). Inhibiting myosin light chain kinase induces apoptosis in vitro and in vivo. Mol. Cell. Biol..

[32] Soriano O., Alcón-Pérez M., Vicente-Manzanares M., Castellano E. (2021). The Crossroads between RAS and RHO Signaling Pathways in Cellular Transformation, Motility and Contraction. Genes.

[33] Pu Y., Huang L., Prins G.S. (2004). Sonic hedgehog-patched Gli signaling in the developing rat prostate gland: Lobe-specific suppression by neonatal estrogens reduces ductal growth and branching. Dev. Biol..

[34] Bieberich C.J., Fujita K., He W.W., Jay G. (1996). Prostate-specific and androgen-dependent expression of a novel homeobox gene. J. Biol. Chem..

[35] Sciavolino P.J., Abrams E.W., Yang L., Austenberg L.P., Shen M.M., Abate-Shen C. (1997). Tissue-specific expression of murine Nkx3.1 in the male urogenital system. Dev. Dyn..

[36] Ho S.M., Damassa D., Kwan P.W., Seto H.S., Leav I. (1985). Androgen receptor levels and androgen contents in the prostate lobes of intact and testosterone-treated Noble rats. J. Androl..

[37] Shain S.A., Boesel R.W. (1977). Aging-associated diminished rat prostate androgen receptor content concurrent with decreased androgen dependence. Mech. Ageing Dev..

[38] Prins G.S. (1989). Differential regulation of androgen receptors in the separate rat prostate lobes: Androgen independent expression in the lateral lobe. J. Steroid Biochem..

[39] Prins G.S., Woodham C. (1995). Autologous regulation of androgen receptor messenger ribonucleic acid in the separate lobes of the rat prostate gland. Biol. Reprod..

[40] Kling D.E., Lorenzo H.K., Trbovich A.M., Kinane T.B., Donahoe P.K., Schnitzer J.J. (2002). MEK-1/2 inhibition reduces branching morphogenesis and causes mesenchymal cell apoptosis in fetal rat lungs. Am. J. Physiol.-Lung Cell. Mol. Physiol..

[41] Fisher C.E., Michael L., Barnett M.W., Davies J.A. (2001). Erk MAP kinase regulates branching morphogenesis in the developing mouse kidney. Development.

[42] Somlyo A.V., Bradshaw D., Ramos S., Murphy C., Myers C.E., Somlyo A.P. (2000). Rho-kinase inhibitor retards migration and in vivo dissemination of human prostate cancer cells. Biochem. Biophys. Res. Commun..

[43] Somlyo A.V., Phelps C., Dipierro C., Eto M., Read P., Barrett M., Gibson J.J., Burnitz M.C., Myers C., Somlyo A.P. (2003). Rho kinase and matrix metalloproteinase inhibitors cooperate to inhibit angiogenesis and growth of human prostate cancer xenotransplants. FASEB J..

[44] Moore K.A., Polte T., Huang S., Shi B., Alsberg E., Sunday M.E., Ingber D.E. (2005). Control of basement membrane remodeling and epithelial branching morphogenesis in embryonic lung by Rho and cytoskeletal tension. Dev. Dyn..

[45] Madueke I., Hu W.Y., Hu D., Swanson S.M., Vander Griend D., Abern M., Prins G.S. (2019). The role of WNT10B in normal prostate gland development and prostate cancer. Prostate.

[46] Madueke I.C., Hu W.Y., Huang L., Prins G.S. (2018). WNT2 is necessary for normal prostate gland cyto-differentiation and modulates prostate growth in an FGF10 dependent manner. Am. J. Clin. Exp. Urol..

[47] Schlessinger K., Hall A., Tolwinski N. (2009). Wnt signaling pathways meet Rho GTPases. Genes Dev..

[48] Habas R., Kato Y., He X. (2001). Wnt/Frizzled activation of Rho regulates vertebrate gastrulation and requires a novel Formin homology protein Daam1. Cell.

[49] Shi W., Xu C., Gong Y., Wang J., Ren Q., Yan Z., Mei L., Tang C., Ji X., Hu X. (2021). RhoA/Rock activation represents a new mechanism for inactivating Wnt/β-catenin signaling in the aging-associated bone loss. Cell Regen..

[50] Aaron L., Franco O.E., Hayward S.W. (2016). Review of Prostate Anatomy and Embryology and the Etiology of Benign Prostatic Hyperplasia. Urol. Clin. N. Am..

[51] Prins G.S., Hu W.Y., Xie L., Shi G.B., Hu D.P., Birch L., Bosland M.C. (2018). Evaluation of Bisphenol A (BPA) Exposures on Prostate Stem Cell Homeostasis and Prostate Cancer Risk in the NCTR-Sprague-Dawley Rat: An NIEHS/FDA CLARITY-BPA Consortium Study. Environ. Health Perspect..

[52] Prins G.S., Ye S.H., Birch L., Zhang X., Cheong A., Lin H., Calderon-Gierszal E., Groen J., Hu W.Y., Ho S.M. (2017). Prostate Cancer Risk and DNA Methylation Signatures in Aging Rats following Developmental BPA Exposure: A Dose-Response Analysis. Environ. Health Perspect..

