# Supplementary material for: Morphometric Analysis of Rat Prostate Development: Roles of MEK/ERK and Rho Signaling Pathways in Prostatic Morphogenesis
Source: Biomolecules. 2021 Dec 4;11(12):1829. doi: 10.3390/biom11121829 (PMC8698940; doi:10.3390/biom11121829)
Supplement: Supplementary file 1 [file biomolecules-11-01829-s001.zip › supplementary.pdf]

Supplemental video

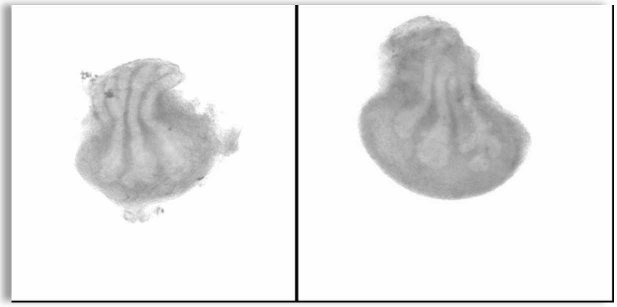

Scheme 20. μM U0126 over 90 hr. Representative videos from six separate sets of experiments are shown.

Supplemental figures

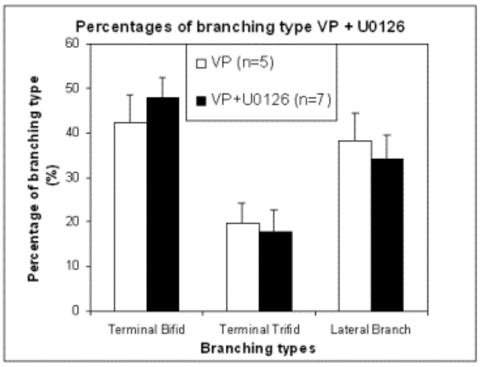

Figure S1. Percentage of branching events occurring as terminal bifeds, terminal trifeds or lateral side branches in VPs cultured in the absence or presence of 20 μM U0126. Bars represent the mean ± SEM for 5–7 sets of experiments.

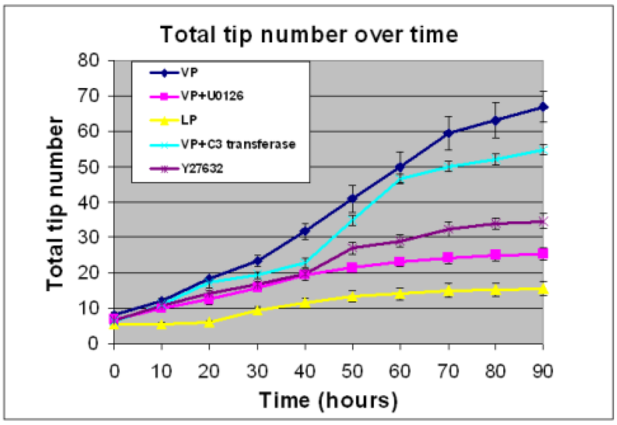

Figure S2. Total tip number in 3D analysis as a function of time in rat contralateral pnd 0 untreated VPs (dark blue), 20 μM U0126 (red), C3-transferase (light blue) and 20 μM Y-27632 treated VPs (purple) as well as LPs (yellow) cultured for 90 hr. Each data point represents the mean of 4 sets of experiments and bars denote SEM.

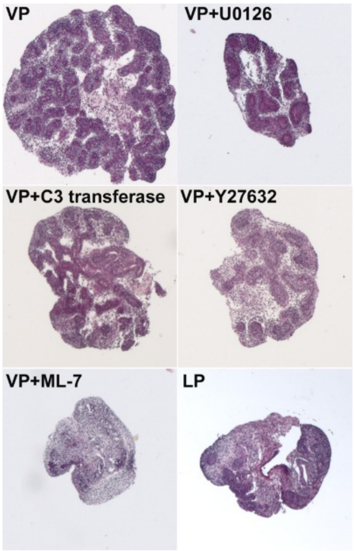

Figure S3. Representative images of H&E staining from cultured VPs with different treatments and LP cross sections.
